# Detection of Hate Speech in COVID-19–Related Tweets in the Arab Region: Deep Learning and Topic Modeling Approach

**DOI:** 10.2196/22609

**Published:** 2020-12-08

**Authors:** Raghad Alshalan, Hend Al-Khalifa, Duaa Alsaeed, Heyam Al-Baity, Shahad Alshalan

**Affiliations:** 1 King Saud University Riyadh Saudi Arabia

**Keywords:** COVID-19, coronavirus, Twitter, hate speech, social network analysis, social media, public health, pandemic, deep learning, non-negative matrix factorization, NMF, convolutional neural network, CNN

## Abstract

**Background:**

The massive scale of social media platforms requires an automatic solution for detecting hate speech. These automatic solutions will help reduce the need for manual analysis of content. Most previous literature has cast the hate speech detection problem as a supervised text classification task using classical machine learning methods or, more recently, deep learning methods. However, work investigating this problem in Arabic cyberspace is still limited compared to the published work on English text.

**Objective:**

This study aims to identify hate speech related to the COVID-19 pandemic posted by Twitter users in the Arab region and to discover the main issues discussed in tweets containing hate speech.

**Methods:**

We used the ArCOV-19 dataset, an ongoing collection of Arabic tweets related to COVID-19, starting from January 27, 2020. Tweets were analyzed for hate speech using a pretrained convolutional neural network (CNN) model; each tweet was given a score between 0 and 1, with 1 being the most hateful text. We also used nonnegative matrix factorization to discover the main issues and topics discussed in hate tweets.

**Results:**

The analysis of hate speech in Twitter data in the Arab region identified that the number of non–hate tweets greatly exceeded the number of hate tweets, where the percentage of hate tweets among COVID-19 related tweets was 3.2% (11,743/547,554). The analysis also revealed that the majority of hate tweets (8385/11,743, 71.4%) contained a low level of hate based on the score provided by the CNN. This study identified Saudi Arabia as the Arab country from which the most COVID-19 hate tweets originated during the pandemic. Furthermore, we showed that the largest number of hate tweets appeared during the time period of March 1-30, 2020, representing 51.9% of all hate tweets (6095/11,743). Contrary to what was anticipated, in the Arab region, it was found that the spread of COVID-19–related hate speech on Twitter was weakly related with the dissemination of the pandemic based on the Pearson correlation coefficient (r=0.1982, *P*=.50). The study also identified the commonly discussed topics in hate tweets during the pandemic. Analysis of the 7 extracted topics showed that 6 of the 7 identified topics were related to hate speech against China and Iran. Arab users also discussed topics related to political conflicts in the Arab region during the COVID-19 pandemic.

**Conclusions:**

The COVID-19 pandemic poses serious public health challenges to nations worldwide. During the COVID-19 pandemic, frequent use of social media can contribute to the spread of hate speech. Hate speech on the web can have a negative impact on society, and hate speech may have a direct correlation with real hate crimes, which increases the threat associated with being targeted by hate speech and abusive language. This study is the first to analyze hate speech in the context of Arabic COVID-19–related tweets in the Arab region.

## Introduction

Social media platforms such as Twitter provide valuable venues for information sharing, communication, and knowledge production. However, these platforms have also been increasingly exploited for the propagation of hate speech. Hate speech can be generally defined as language that aims to target a specific group on the basis of characteristics such as ethnic origin, religion, or gender [1].

Hate speech on the internet is a complex concept with a wide spectrum of targets, forms, and other related concepts [2,3]. While there is no formal definition of hate speech, there is general agreement among scholars and service providers to define it as any language that attacks a person or a group based on a characteristic such as race, color, ethnicity, gender, sexual orientation, nationality, or religion [3]. This type of discriminatory and hateful speech can have a destructive impact on society, as it threatens the culture of coexistence and unity. It is also evident that hate speech has a strong connection with actual hate crimes [3,4], which increases the risks associated with being targeted by hate speech and abusive language.

In mid-February 2020, the COVID-19 virus pandemic started to penetrate the Arab region physically and virtually, and Arab social media users started talking about the spread of the disease using different platforms. The pandemic became a trending topic that was visible to all users on Twitter within the Arab region. Since then, multiple voices have been raised across social media using the COVID-19 pandemic as a vehicle for spreading hate speech.

The use of social media during pandemics for a variety of public health purposes has been demonstrated in a growing body of literature. One systematic review on social media and emerging infectious diseases identified three major approaches, namely assessment of public opinion, social media use by organizations, and evaluation of information accuracy [4]. Another systematic review on studies that primarily use web-based social networks for pandemic detection and tracking found that social networks have rich information that can be utilized to develop an almost real-time pandemic surveillance system [5]. Other studies have used Twitter and other sources of data to build a surveillance system [6] and develop automatic methods for quantifying the scientific quality and sensationalism of individual news records during the pandemic [7].

During the unfolding COVID-19 pandemic, many research studies have focused on Twitter to characterize the impact of the pandemic on public responses and behaviors. One of these studies analyzed the main topics discussed by Twitter users during the crisis by leveraging techniques such as latent Dirichlet allocation (LDA) and sentiment analysis [8]. Another study found that frequent use of social media during the pandemic contributed to information overload, which had a significant impact on individuals’ coping perceptions [9].

In addition, web-based social question-and-answer forums have been analyzed to identify topic communities and asses the appropriateness of the answers during the early stage of the COVID-19 outbreak [10]. Other studies have analyzed Twitter data to understand the impact of COVID-19 on specific public attitudes and behaviors, such as xenophobia [11] and the spread of the 5G conspiracy theory [12]. As discussed in [11], the pandemic has triggered discrimination and stigma toward specific groups on social media. This phenomenon was further explored in [13], with a specific focus on building an automatic tool for detecting hate speech in Spanish tweets related to newspaper articles about the COVID-19 pandemic. The preliminary results showed that 9% of the tweets contained hate speech.

As can be seen, the massive scale of social media platforms requires an automatic solution for detecting hate speech. Such solutions will help reduce the need for manual analysis of content. Most previous literature has cast the hate speech detection problem as a supervised text classification task using classical machine learning methods or, more recently, deep learning methods. However, studies investigating this problem in Arabic cyberspace are still limited compared to the published work on English text.

The aim of this study is to automatically identify hate speech posted by Arabic-speaking Twitter users related to the COVID-19 pandemic using a deep learning approach. We opted to analyze the spread of hate speech on the Twitter platform given its increasing popularity among Arab users [14]. According to [15], nearly 9 out of 10 Arab adults use the internet, and 22% of them use the Twitter platform actively. In Saudi Arabia alone, there are more than 10 million active Twitter users, akin to almost 38% of the population.

Our research objectives are to answer the following research questions: (1) In which Arab country were the most COVID-19–related hate tweets posted during the pandemic? (2) When were the highest number of hate tweets posted in the Arab region during the pandemic? (3) Does the increase in the number of COVID-19 cases or deaths coincide with an increase in the number of hate tweets? and (4) What are the main topics and issues being discussed in hate tweets during the COVID-19 pandemic?

The rest of the paper is structured as follows: the Methods section presents our methods, including a description of the neural network models and the preprocessing steps. In the next sections, we provide the results and discuss them. Finally, we conclude our work and discuss future directions.

## Methods

**Figure 1:**
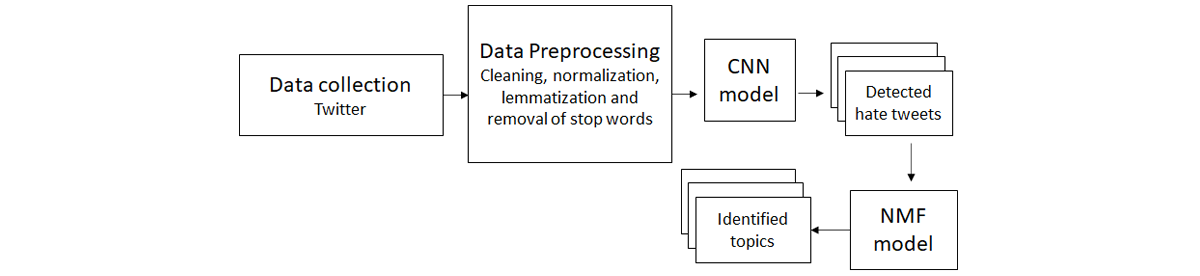
Methodology workflow. CNN: convolutional neural network; NMF: utilized nonnegative matrix factorization.

In this study, we aimed to automatically detect hate tweets posted by Twitter users in the Arab region during the COVID-19 pandemic using a deep learning approach and topic modeling. The workflow of our methodology is depicted in Figure 1. In the following subsections, we describe each step in more detail.

### Data Collection

We used the ArCOV-19 [16] dataset, which is an ongoing collection of Arabic tweets related to COVID-19 starting from January 27, 2020. The tweets were collected using the Twitter search application programming interface (API) to obtain the most popular tweets using different queries, such as simple keywords (eg, *Corona*), hashtags (eg, *#Corona*), or phrases (eg, *novel Coronavirus*). The search was customized to exclude retweets and non-Arabic tweets. At the time of writing this paper, the ArCOV-19 dataset contained almost 1 million popular tweets (ie, tweets that received the maximum social interactions and impressions) collected from January 27 to April 30, 2020. In compliance with the Twitter content redistribution policy, the authors of the ArCOV-19 dataset only released the tweet IDs. Therefore, we recollected the full tweet objects using the *Twarc* library [17], which is a Python library for retrieving Tweets as JavaScript Object Notation (JSON) objects using the Twitter API based on the given IDs. We started the preprocessing pipeline by removing retweets and duplicate tweets that had identical content to reduce the effect of malicious tweets posted by social bots. After that, and due to the inaccessibility of some tweets (deleted tweets or deactivated accounts), the total number of tweets we retrieved was reduced to 975,316.

### Data Preprocessing

The retrieved JSON object for each tweet is a mix of many attributes. For this study, we retained the following attributes: tweet ID, tweet text, creation time, user ID, and location. The location field represents the location profile metadata as declared by the Twitter user. However, this field contains free-form text entered manually by the user; hence, this field can be noisy, as users can type anything. Therefore, we needed to resolve the text to an exact location (in our case, a country).

The procedure to extract the country from the location text was as follows. First, for each country in the Arab region, we prepared a list that contains the country names and all possible city names derived from GeoNames [18], a geographical database that contains over 25 million geographical names. After that, each list was translated into Arabic and automatically extended to include different morphological variant forms of the same word. Also, each list was manually modified to include more informal terms and forms that Twitter users frequently use to refer to their countries or cities. For example, Emirati users might type “Dar Zayed” in their location field instead of “United Arab Emirates” or “UAE”, and Kuwaiti users usually write “Q8” instead of “Kuwait”. Also, to minimize the mismatching problem between the prepared list and the location field, we normalized the names in our lists and the location field to reduce orthographical variations; for example, different forms of ”
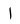
” (“
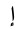
”, “
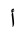
”, and “
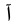
”) were replaced with “
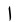
”, while the letter “
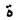
” was replaced with “
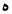
” and the letter “
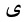
” was replaced with “
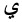
”. After that, we performed several cleaning and denoising steps on the lists and location fields, including the removal of punctuation marks, additional white spaces, and diacritics. Then, to map each text location to a specific country, we checked if at least one of the words in the location text appeared in the list for any country. If it did, the location was resolved to that specific country. For example, if a user typed “Riyadh”, “Najd”, “Jeddah”, or “Saudi”, we resolved this location to “Saudi Arabia.” Finally, we dropped all tweets that belonged to users with no detected location.

To prepare the tweets to be fed as inputs into the convolutional neural network (CNN) classification model and the nonnegative matrix factorization (NMF) model, we firstly applied several preprocessing steps to the text of the tweets. First, we removed punctuation, consecutive words, additional white spaces, and non-Arabic letters. We also replaced emojis with their descriptions for CNN inputs and removed them for the NMF model. We also normalized Twitter-specific tokens by converting the links and mentions to “URL” and “mention,” respectively. After that, various forms of the same word were lemmatized by converting them to the main word using the Farasa tool [19]; finally, we removed stop words from the text.

### Hate Speech Detection Using the CNN Model

The processed tweets were analyzed for hate speech using the pretrained CNN model. The CNN model used in this study is based on our previous work [20] in which we addressed the problem of hate speech spread in the Arabic Twittersphere. In this study, a CNN was trained on almost 10,000 tweets that were manually labeled as hate tweets or non–hate tweets. The model contained five layers: an input layer (embedding layer), a convolution layer, which basically consists of 3 parallel convolution layers with different kernel sizes (2, 3, and 4), a pooling layer, a hidden dense layer, and a final output layer that assigned a “hate score” (ranging from 0 to 1) to each text. The results showed that the CNN model achieved the best performance (compared to other models), with an F1 score of 79% and accuracy of 83%. Therefore, we used it in this study.

The analysis was performed on the tweet texts, and each tweet was given a score between 0 and 1, with 1 being the most hateful text. We used the default threshold of 0.5 (or 50%) to classify a prediction with a probability of 0.5 or more as a hate tweet and any value less than 0.5 as a non–hate tweet. We further classified hate prediction into three levels based on score: low (0.50-0.67), average (0.68-0.85), and high (0.86-1.00). The hate tweets were then categorized into three levels (low, colored yellow; average, colored orange; and high, colored red) based on the detected hate scores.

### Topic Modeling Using NMF

NMF is an unsupervised approach for reducing the dimensionality of nonnegative matrices [21], and it has been widely used to discover the underlying relationships between texts and identify latent topics [22,23]. In this study, we opted to employ NMF to discover the semantic structure and discussed topics within the set of hate tweets. We particularly chose NMF given its ability to produce more coherent topics than other popular topic modeling techniques such as the LDA model, as shown in this study. We used the sklearn library [24] to implement the NMF model. To apply NMF, we first preprocessed the tweets as described earlier. Then, we created the features by transforming the processed tweets into term frequency–inverse document frequency vectors (unigrams and bigrams).

## Results

### Model Results

As mentioned, the first step in the hate speech analysis was to analyze the processed tweets using the pretrained CNN model. Table 1 presents the total numbers of detected hate tweets and non–hate tweets in the Arab region, along with the three levels of hate (low, average, and high). It can be observed in Table 1 that 535,811 of the 547,554 collected tweets related to the COVID-19 pandemic (97.8%) could be classified as non–hate tweets, while 11,743 (3.2%) were classified as hate tweets. Generally, most of the hate tweets fell in the low level range (8385/11,743, 71.4%). On the other hand, only 2.89% of hate tweets (340/11,743) were categorized as high level.

**Table 1:** Total numbers of hate tweets and non–hate tweets in the Arab region during the period of study (N=547,554).

Type of tweet		Number of tweets, n (%)
Non–hate tweets		535,811 (97.8)
Hate tweets		11,743 (3.2)
**Hate levels^a^ (n=11,743)**		
	Low	8385 (71.4)
	Average	3018 (25.7)
	High	340 (2.9)

^a^Based on scores assigned by the convolutional neural network model (low: 0.50-0.67; average: 0.68-0.85; high: 0.86-1.00).

### Descriptive Statistical Analysis

To obtain more insight into the obtained results, we analyzed the hate tweets from two different aspects: by country and by time period. In addition, we obtained the WHO COVID-19 statistics (number of cases and deaths) for each country and during the aforementioned time periods.

#### By Country

**Figure 2:**
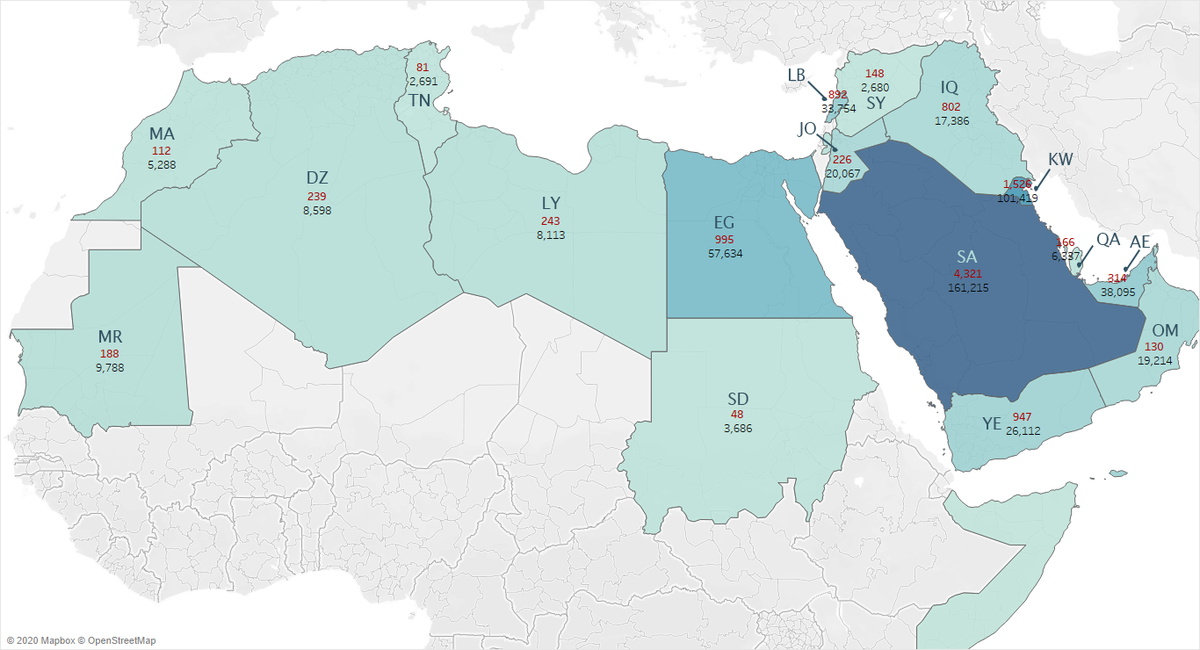
Number of hate tweets (red) and total tweets (black) in each Arab country, where a darker color depicts a higher number of hate tweets in that country (MA: Morocco, MR: Mauritania, DZ: Algeria, TN: Tunisia, LY: Libya, EG: Egypt, JO: Jordan, LB: Lebanon, SY: Syria, IQ: Iraq, SA: Saudi Arabia ,YE: Yemen, KW: Kuwait, QA: Qatar, AE: United Aran Emirates, OM: Oman).

The map in Figure 2 displays the number of hate tweets and their distribution across the Arab countries, where the number of hate tweets is shown in red, the number of total tweets is shown in black, and a dark color depicts a high number of hate tweets in that country.

Table 2 compares the number of hate tweets in Arab countries and the distribution of hate tweets with different hate levels (low, average, and high). Out of the 22 Arab countries, the 10 most Twitter-active countries with respect to the number of COVID-19–related tweets are shown in the table. The results of the other 12 countries are combined in one column, titled “Other Arab countries.” In addition, Table 2 presents COVID-19 statistics (number of cases and deaths) for each country. It can be observed from Table 2 that country-wise, most COVID-19 tweets are of the non-hate class, and the percentages of hate tweets are considerably low. For instance, Saudi Arabia, which is the most Twitter-active Arab country as stated in the Digital 2020 Global Report [25], has the highest number of COVID-19–related tweets (165,536 tweets), of which only 4321 (2.61%) were classified as hate tweets.

In line with our previous observation, where most of the hate tweets were at the low level of hate, we found that the largest numbers of hate tweets were at the low hate level. For example, 2813 of the 4321 hate tweets in Saudi Arabia (65.10%) fell in the low level range, while 200 (4.63%) were at a high level. Similar results can be seen when considering Mauritania, which has the smallest number of COVID-19–related tweets among the top 10 Arab countries. 81.4% of hate tweets (153/188) in Mauritania are at the low hate level, and only 1.1% (2/188) are at a high level.

**Table 2:** Statistics of COVID-19–related hate tweets posted in Arab countries.

Variable		Country										
		Saudi Arabia	Kuwait	Egypt	UAE^a^	Lebanon	Yemen	Jordan	Oman	Iraq	Mauritania	Other Arab countries^b^
Population (million)		34	4	102	10	7	30	10	5	40	5	187
Posted tweets (N=535,811), n (%)		165,536 (24.2)	102,945 (15.0)	58,629 (8.6)	38,409 (5.6)	34,646 (5.1)	27,059 (4.0)	20,293 (3.0)	19,344 (2.8)	18,188 (2.7)	9976 (1.5)	52,529 (7.1)
Hate tweets (n=11,743), n (%)		4321 (2.6)	1526 (1.5)	995 (1.7)	314 (0.8)	892 (2.6)	947 (3.5)	226 (1.1)	130 (0.7)	802 (4.4)	188 (1.9)	1402 (2.7)
**Hate levels of tweets^c^**												
	Low (n=8385), n (%)	2813 (65.1)	1153 (75.6)	747 (75.0)	245 (78.0)	700 (78.5)	640 (67.5)	160 (70.8)	104 (80.0)	624 (77.8)	153 (81.4)	1046 (74.6)
	Average (n=3018), n (%)	1308 (30.3)	347 (22.7)	224 (22.5)	65 (20.7)	184 (20.6)	277 (29.2)	62 (27.4)	21 (16.6)	168 (20.9)	33 (17.6)	329 (23.5)
	High (n=340), n (%)	200 (4.6)	26 (1.7)	24 (2.4)	4 (1.3)	8 (0.9)	30 (3.2)	4 (1.8)	5 (3.8)	10 (1.2)	2 (1.1)	27 (1.9)
	Average hate level score	0.643	0.613	0.617	0.606	0.605	0.634	0.623	0.602	0.610	0.602	0.623
**COVID-19 statistics (n)**												
	Cases (n=78,037)	22753	4024	5537	12481	725	6	453	2348	2003	7	27700
	Deaths (n=1593)	162	26	392	105	24	0	8	11	92	1	772

^a^UAE: United Arab Emirates.

^b^Other Arab countries: this column combines the results of the remaining Arab countries (Palestine, Algeria, Libya, Bahrain, Morocco, Qatar, Sudan, Syria, Tunisia, Comoros, and Somalia).

^c^Based on scores assigned by the convolutional neural network model (low: 0.50-0.67; average: 0.68-0.85; high: 0.86-1.00).

**Figure 3:**
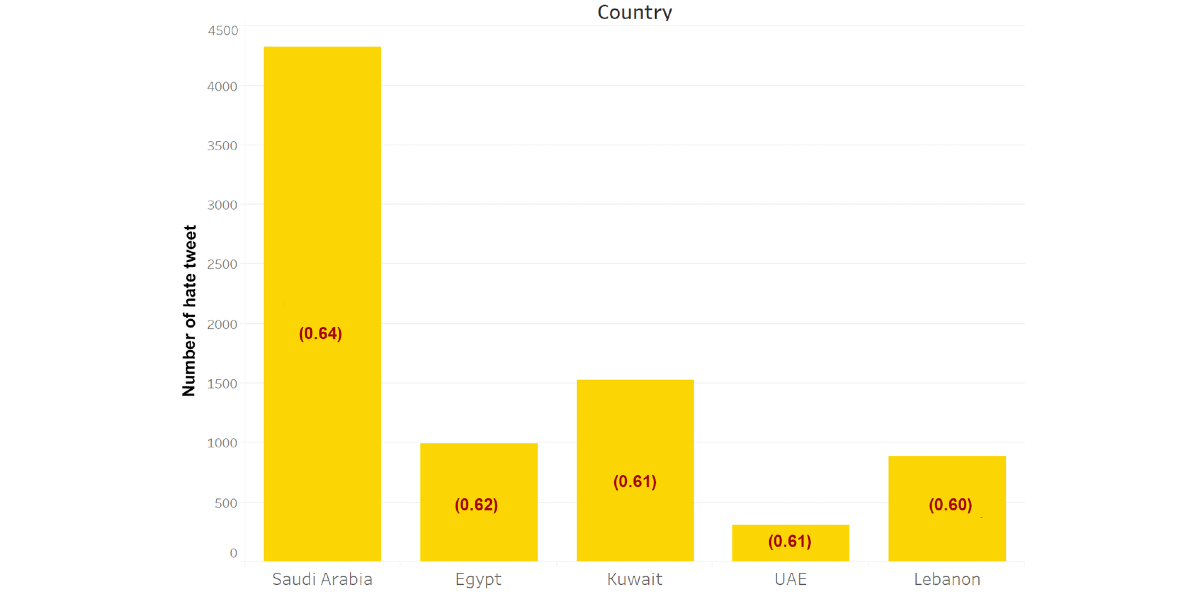
Number of COVID-19–related hate tweets per country with the average hate level scores in brackets (low: 0.50-0.67; average: 0.68-0.85; high: 0.86-1.00). UAE: United Arab Emirates.

Figure 3 illustrates the statistics of hate tweets for the top five Arab countries with respect to number of COVID-19–related tweets, showing the average hate level of all hate tweets in red. It can be seen from Figure 3 that average hate level for the countries shown falls in the low hate range, which is coded in yellow. This strongly supports our first finding that the majority of hate tweets do not contain a high level of hate.

#### By Time Period

The ArCOV-19 [15] data used in our study cover the period of January 27 to April 30, 2020. This period was divided into three specific time durations: January 27 to February 29, March 1-30, and April 1-30. Table 3 includes the number of COVID-19–related tweets in the Arab region during each time period, together with the number of hate tweets and their distribution in the three different hate levels. It also presents the number of COVID-19 cases and deaths in each time period.

**Table 3:** Statistics of COVID-19–related hate tweets per time period (N=547,554).

Variable		Time period (2020)		
		January 27-February 29	March 1-30	April 1-30
Total tweets (N=547,554), n (%)		118,991 (21.7)	253,806 (46.4)	174,757 (31.9)
Hate tweets (n=11,743), n (%)		3014 (25.7)	6095 (51.9)	2634 (22.4)
**Hate levels of tweets^a^**				
	Low	2198 (72.9)	4300 (70.5)	1887 (71.6)
	Average	741 (24.6)	1617 (26.5)	660 (25.1)
	High	75 (2.5)	178 (2.9)	87 (3.3)
	Average hate level score	0.622	0.628	0.625
**COVID-19 statistics (n)**				
	Cases	133	7447	70,092
	Deaths	0	202	1350

^a^Based on scores assigned by the convolutional neural network model (low: 0.50-0.67; average: 0.68-0.85; high: 0.86-1.00).

From Table 3, it can be clearly noted that after the end of the first period (January 27 to February 29), the number of COVID-19–related tweets spiked, with an increase of 113.3% compared to the first period. However, the number then decreases by 31.1% in the third period (April 1-30). Likewise, the number of hate tweets increased by 102.2% in the second period, then decreased by 56.8% in the third period. In contrast, the number of identified COVID-19 cases continued to grow during all three periods.

Looking at the levels of hate tweets per time period, it can be seen that although the number of hate tweets falling in the high level range is extremely small compared to those in the low level range, the high-level hate tweets have a tendency to increase with increasing number of COVID-19 cases and deaths, showing percentages of 2.49% (75/3014), 2.9% (178/6095), and 3.3% (87/2634) for the first, second, and third periods, respectively. On the other hand, the low and average hate levels do not follow the same behavior.

**Figure 4:**
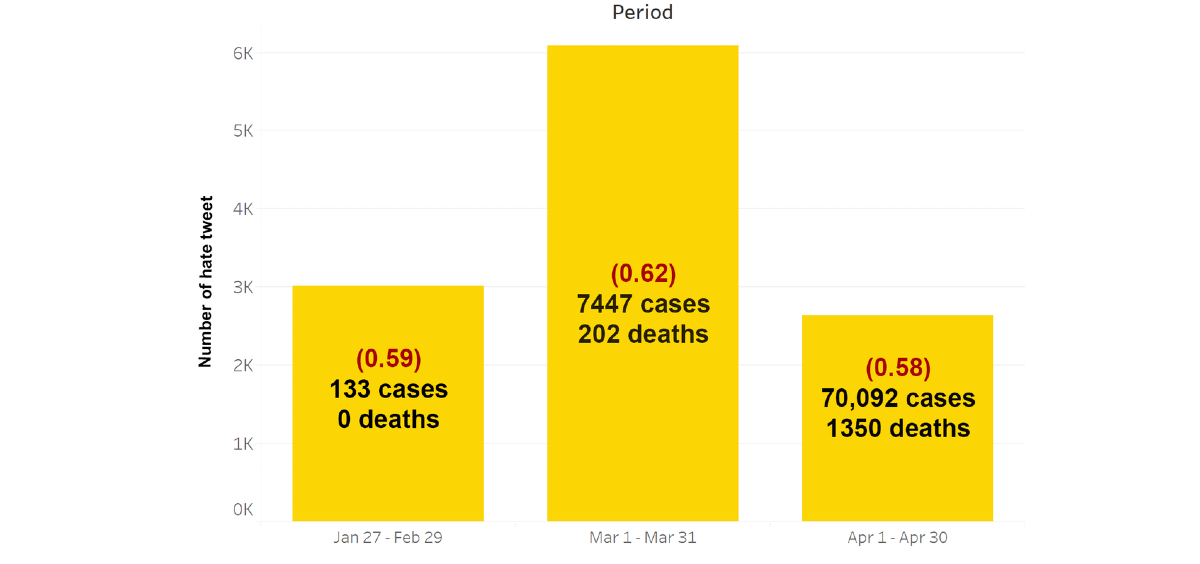
Numbers of hate tweets and numbers of COVID-19 cases and deaths per time period with the average hate level scores in brackets (low: 0.50-0.67; average: 0.68-0.85; high: 0.86-1.00).

Figure 4 illustrates the statistics of the number of hate tweets per time period and the numbers of COVID-19 cases and deaths together with the average hate levels of all hate tweets (shown in red). It can be observed from Figure 4 that the average hate levels for the three time periods are in the low-level range, which is in line with our initial finding that the majority of hate tweets do not reach the high level of hate.

From the previous results, it can be seen that there is a weak link between the number of hate tweets and the spread of COVID-19 per country or time period. This is because the number of hate tweets does not follow the same pattern of the number of COVID-19 cases, as illustrated in Table 2 and Table 3. To investigate this further, we tested the correlations between the number of COVID-19–related hate tweets and the number of COVID-19 cases in the data of 21 Arab countries during the 3 time periods under study. Using the Pearson correlation test [26], we obtained an *r* value of 0.1982 at *P*=.50. Although *r* is technically positive, the relationship between the number of hate tweets and the number of COVID-19 cases is weak (the nearer the value of *r* to zero, the weaker the relationship [26]).

### NFM Results

To understand the main topics discussed by Arab users during the pandemic, we employed NFM as discussed earlier in this paper. We extracted 7 topics from the hate tweets. The distributions and examples of the top terms associated with each topic are shown in Table 4. As shown in Table 4, the most relevant terms for Topic 1 were terms that were usually used to criticize China as a country for being the source of SARS-CoV-2, the virus that cause COVID-19. Within the same theme, tweets in Topic 4 expressed hate speech toward Chinese people and their eating habits as being responsible for spreading COVID-19 worldwide. China was also the target of attack in Topic 5, in which many users expressed their belief that COVID-19 served as a “divine punishment against China” because of the Uyghur-Chinese conflict.

**Table 4:** Identified topics in hate tweets and examples of the most common words in each topic.

Topic number	Main theme	Examples of the top unigrams and bigrams	Distribution
1	China and COVID-19	Cursed, China, curse China, Curses on, life, new	4.3%
		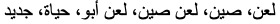	
2	Iran as a source of COVID-19	Export, Gulf, country, Terrorism, disease, spread	5.07%
		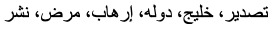	
3	Saudi citizens visiting Iran	Saudi Arabia, Saudi, Bahrain, citizen, travel, passport	9.44%
		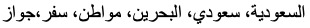	
4	Chinese eating habits and COVID-19	Dog, animal, bat, eating, meat, snake	5.72%
		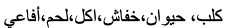	
5	China and Uighur	China, Muslim, Uyghur, Chinese, pig, punishment	7.38%
		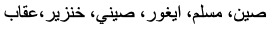	
6	Iran regime	Regime, Mullahs, Iran, Iranian People, Tehran, outbreak	5.60%
		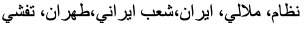	
7	General political tweets, conspiracies, COVID-19 as an exaggerated threat	Country, people, disease, party, Iraq, Egypt	62.45%
		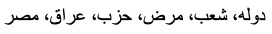	

On the other hand, Topic #2 contained several tweets accusing Iran of deliberately spreading COVID-19 to the Gulf Cooperation Council (GCC) countries. Topic #6 is also about Iran and COVID-19; however, the tweets in this topic were more political, attacking the Iranian regime and its politics. Topic #3 included cases of hate speech against Saudi citizens who visited Iran during the pandemic as being responsible for the spread of COVID-19 in Saudi Arabia.

Finally, in Topic #7, we observed that most of the inspected tweets were written in a political context, where the most frequently mentioned countries were Iraq and Egypt. We also observed many tweets spreading conspiracy theories about the scale of the pandemic and its origin, and some tweets stated that COVID-19 is an exaggerated crisis compared to the current regional crises and conflicts.

## Discussion

### Principal Results

This study aimed to address four research questions: (1) In which Arab country were the most COVID-19 hate tweets posted during the COVID-19 pandemic? (2) When were the highest number of hate tweets posted in the Arab region during the pandemic? (3) Does the increase in the number of COVID-19 cases or deaths coincide with an increase in the number of hate tweets? and (4) What are the main topics and issues being discussed in hate tweets during the COVID-19 pandemic?

In regard to the Arab country from which the highest number of hate tweets related to the COVID-19 pandemic originated, Saudi Arabia, which has the highest number of COVID-19–related tweets (165,536 tweets), had the highest number of hate tweets (4321), as shown in Table 2. Therefore, Saudi Arabia is the country in which the most COVID-19 hate tweets were posted during the pandemic. This can be attributed to Saudi Arabia numbering in fourth place in the top 20 countries and the only Arab country in the top 20 based on the largest active Twitter audience worldwide as mentioned in the Digital 2020 Global Report [25].

However, the percentage of hate tweets in Saudi Arabia, which is 2.6% (4321/165,536), is not the highest in the Arab region. This indicates that the number of COVID-19–related tweets does not necessarily reflect the number of hate tweets (as highlighted in Table 2). This can be clearly seen in the case of Yemen, where 27,059 tweets were posted, 3.5% of which were classified as hate tweets (n=947). Yemen having the highest percentage of hate tweets can be linked to the political and security instability in Yemen due to the Yemen Civil War, which began in 2014, and its psychological effect on the citizens of this country.

In addressing the second research question, this paper identifies the time period during which the number of hate tweets was the highest in the Arab region. As shown in Table 3, the second period (March 1-30) has the highest number of hate tweets (6095 tweets), which represents 51.9% of the 11,743 hate tweets in the three-month period of January 27 to April 30, 2020. Despite the fact that the highest numbers of cases and deaths occurred in the third period (April 1-30), the number of hate tweets reached its maximum during the second period. There are two possible explanations for this finding. First, it may be related to the beginning of the spread of COVID-19 in the Arab region, which occurred in March 2020. This period witnessed the highest level of public attention, which can be seen through the high number of Twitter interactions during the pandemic. The other explanation is that people began to adjust to coping with the pandemic and health precautions in the third period, and the severity of their panic and anxiety decreased.

The third research question investigated whether the number of hate tweets coincided with the spread of COVID-19 in the Arab region. As clearly shown in Table 2 and Table 3, the spread of COVID-19 hate tweets does not follow the same trend of the increase or decrease in the number of COVID-19 cases or deaths in any Arab country or during the studied time period. This can be interpreted as a weak relationship between the two variables because the *r* value obtained from the Pearson correlation test was 0.1982 (*P*=.50) [26]. Moreover, it can be observed from Table 3 that most of the hate tweets appeared in the second period, while the numbers of COVID-19 cases and deaths are highest in the third period, with percentages of 90.2% and 87%, respectively.

Regarding the fourth research question, this study sought to identify the topics discussed by Arab Twitter users in hate tweets. We analyzed the 7 topics extracted from the NFM model, and as shown in Table 4, most of the identified topics revolved around two themes: China (topics 1, 4, and 5) and Iran (topics 2, 3, and 6). It can be clearly noted that the COVID-19 pandemic fueled nationalism and ethnic conflict among Arab Twitter users. This spike of hate speech against China and Iran (and Arab individuals who traveled to Iran) can be attributed to the fact that China and Iran were increasingly accused in the media of spreading SARS-CoV-2 worldwide and to Arab countries, respectively. The United Nations recently published a guidance note [27] on addressing and countering COVID-19–related hate speech. The note stated that the pandemic has given rise to a new wave of hate speech and discrimination, which our results clearly confirm. After manual inspection of a few sample tweets from the identified topics, we observed that most of the tweets incited hatred and blame against individuals or groups belonging to certain ethnicities or from certain countries. This type of COVID-19–related hate speech could entail serious consequences, making the targeted groups more vulnerable to violence and discrimination and exposing them to political and social exclusion, isolation, and stigmatization.

### Limitations and Future Work

One strength of this study is that it automatically identified hate tweets posted during the COVID-19 pandemic in the Arab region using deep learning and topic modeling. The model was trained on data from similar domains with similar characteristics (ie, noisy tweets with a high variety of dialectal Arabic). The model was able to identify a total of 11,743 hate tweets, which allowed us to study the phenomenon of hate speech on Twitter. However, some limitations of our study do exist. One limitation is that the used model was trained on a dataset collected in a span of 6 months, and certain events and topics dominated social media at that time. This may have affected the model performance and prevented it from capturing new forms of hate, violence, and racism that could be exacerbated by the COVID-19 pandemic. Additional limitations are the short duration of the study and the chances of misclassification of countries due to self-reporting of users’ locations.

In future research, a follow-up analysis could be conducted to compare the prevalence of hate with other psychological aspects, such as anxiety, fear, and depression, in Twitter data during the COVID-19 pandemic.

### Conclusion

The COVID-19 pandemic poses a significant threat to public health in nations worldwide. This study conducted an analysis of hate speech in Twitter data in the Arab region using deep learning and topic modeling. The analysis revealed that the number of non–hate tweets greatly exceeds the number of hate tweets. We also found that the majority of hate tweets were at a low level of hate. This study has shown that in the Arab region, Yemen is the Arab country from which the highest percentage of COVID-19 hate tweets spread during the pandemic. Moreover, it was noted that the time period of March 1-30 showed the highest number of hate tweets, representing 51.9% of all hate tweets.

Contrary to what was expected, the results in this study showed that the number of hate tweets did not follow the same trend as the increase or decrease in the number of COVID-19 cases or deaths. In fact, it was found that the spread of COVID-19 hate speech on Twitter is weakly related to the dissemination of the pandemic based on the Pearson correlation test.

This study also explored the main topics discussed by Arab users in tweets that were identified as hatful. After analyzing the NMF model results, we found that the COVID-19 pandemic clearly triggered hate speech against China and Iran. We also found that most Arabic hate tweets posted during the pandemic propagated hate speech in a political context, which may have been triggered by the ongoing crisis and political tension in the Arab region.

## Abbreviations

API: application programming interface
CNN: convolutional neural network
GCC: Gulf Corporation Council
JSON: JavaScript Object Notation
LDA: latent Dirichlet allocation
NMF: nonnegative matrix factorization
